# Opportunities for primary and secondary prevention of excess gestational weight gain: General Practitioners' perspectives

**DOI:** 10.1186/1471-2296-12-124

**Published:** 2011-11-04

**Authors:** Paige van der Pligt, Karen Campbell, Jane Willcox, Jane Opie, Elizabeth Denney-Wilson

**Affiliations:** 1 Centre for Physical Activity and Nutrition Research, Deakin University, Melbourne, Australia; 2 General Practitioners Association of Geelong, Geelong, Australia; 3 Centre for Primary Health Care and Equity, University of New South Wales, Sydney, Australia

**Keywords:** General Practitioner, Gestational weight gain, Pregnancy, Qualitative, Antenatal

## Abstract

**Background:**

The impact of excess gestational weight gain (GWG) on maternal and child health outcomes is well documented. Understanding how health care providers view and manage GWG may assist with influencing healthy gestational weight outcomes. This study aimed to assess General Practitioner's (GPs) perspectives regarding the management and assessment of GWG and to understand how GPs can be best supported to provide healthy GWG advice to pregnant women.

**Methods:**

Descriptive qualitative research methods utilising semi - structured interview questions to assess GPs perspectives and management of GWG. GPs participating in shared antenatal care in Geelong, Victoria and Sydney, New South Wales were invited to participate in semi - structured, individual interviews via telephone or in person. Interviews were digitally recorded and transcribed verbatim. Data was analysed utilising thematic analysis for common emerging themes.

**Results:**

Twenty eight GPs participated, 14 from each state. Common themes emerged relating to awareness of the implications of excess GWG, advice regarding weight gain, regularity of gestational weighing by GPs, options for GPs to seek support to provide healthy lifestyle behaviour advice and barriers to engaging pregnant women about their weight. GPs perspectives concerning excess GWG were varied. They frequently acknowledged maternal and child health complications resulting from excess GWG yet weighing practices and GWG advice appeared to be inconsistent. The preferred support option to promote healthy weight was referral to allied health practitioners yet GPs noted that cost and limited access were barriers to achieving this.

**Conclusions:**

GPs were aware of the importance of healthy GWG yet routine weighing was not standard practice for diverse reasons. Management of GWG and perspectives of the issue varied widely. Time efficient and cost effective interventions may assist GPs in ensuring women are supported in achieving healthy GWG to provide optimal maternal and infant health outcomes.

## Background

The prevalence of overweight and obesity are increasing among women of childbearing age [1], mirroring rising rates of these conditions in the general population. In the Australian context an estimated 34% of the Australian obstetric population have a body mass index (BMI) greater than 25 kg/m^2^[2]. Similar rates are seen in the United Kingdom with 25% of women overweight and over 15% obese during the first trimester of their pregnancy [3]. In the United States 60% of mothers begin their pregnancy overweight or obese [4]. Some evidence suggests that excess GWG is more common in women who begin their pregnancy at a BMI higher than the normal range [5]. Mean GWGs have increased in developed countries over recent decades [6,7] and the impact of excess gestational weight has gained increased attention. There are currently no Australian recommendations for GWG, however the US Institute of Medicine (IOM) 2009 guidelines [8] are commonly used in practice (see Table 1).

**Table 1:** Institute of Medicine (IOM) 2009 guidelines for total gestational weight gain

Pre pregnancy Weight category	Pre pregnancy BMI	Recommended pregnancy weight gain based on IOM guidelines
Underweight	< 18.5 kg/m^2^	12.5 - 18 kg
Normal weight	18.5 - 24.9 kg/m^2^	11.5 - 16 kg
Overweight	25 - 29.9 kg/m^2^	7 - 11.5 kg
Obese	≥30 kg/m^2^	5 - 9 kg

Excess weight gain during pregnancy places both mother and child at increased risk of serious health complications [9-12] and has been linked with increased risk of caesarean section,[13], gestational hypertension and augmentation of labour [14], preeclampsia [15] and gestational diabetes mellitus [16]. There appears to be a higher risk for adverse pregnancy outcomes when excess GWG is combined with high pre pregnancy maternal BMI. Excess GWG is also associated with increased and persistent postpartum overweight [17,18] which in turn may impact on early termination of breastfeeding [19], on weight trajectories for subsequent pregnancies, and on later BMI [20].

Emerging evidence suggests that excess GWG is associated with increased offspring obesity. In addition to excess GWG, multiple studies have shown that maternal obesity is also a major risk factor for childhood obesity [21-23]. Increased child adiposity has been correlated with excess GWG in neonates and young children [24], in adolescence [25] and in adults at 21 years of age [26]. The high prevalence of child overweight and obesity, coupled with the knowledge that child weight tracks strongly through life prompts us to better understand all opportunities to promote healthy child weight. Understanding how and why clinicians and other health care providers might incorporate management of GWG into their antenatal care is important in maximising prevention of excess GWG during this opportune period.

While the delivery of prenatal care will differ across countries, a number of health professionals, including physicians, midwives and obstetricians consistently deliver such services throughout pregnancy [27]. In Australia, women often choose to have their antenatal care shared between their GP and Obstetrician, known as 'shared care'. With many occasions to engage women at this time, clear opportunities to support women to achieve positive lifestyle changes that may promote healthy GWGs exist. While these health care providers are likely to be central in promoting such changes, little is known about the ways in which such professionals engage on these issues across pregnancy [28].

In particular, General Practitioners (GPs) have been identified as vital contributors to the treatment of overweight and obesity in the non - pregnant population [29,30] and have elsewhere been described as "gatekeepers to the health system", with the opportunity to play a key role in addressing obesity within consultations [31]. Given women will be seeking advice from their healthcare provider frequently during pregnancy and given that GWG is a common and natural progression of pregnancy, early support is likely to be important. The first antenatal visit is often to the GP [32] and hence GPs may provide a key opportunity to influence GWG. General Practitioners specifically participating in shared antenatal care have frequent contact with women throughout their pregnancies and offer specialised obstetric care up until the labour. As such their perspectives on managing gestational weight gain are highly relevant and may help to inform future antenatal practice.

Few studies exist focusing on the views of antenatal healthcare professionals regarding gestational weight management and impact [33] and, to the authors' knowledge, no studies have focused on GPs' perspectives regarding opportunities for prevention of excess GWG. Understanding the ways in which health care providers currently view and manage gestational weight is fundamental to realising this potential. Therefore, the aims of this study were to 1) document the experience of GPs in the assessment and management of women entering pregnancy already overweight, 2) to assess GPs' perceived role in the promotion of healthy GWG more broadly and 3) to understand GPs' views regarding the ways in which they may be supported to promote healthy GWG.

## Methods

### Participants

General Practitioners in Geelong participating in shared antenatal care were identified by telephoning all medical practices from a practitioner list provided by the GP Association of Geelong. Information was obtained regarding whether or not the GP was a participant of shared antenatal care. In Sydney, two divisions of general practice were contacted from where a list of GPs participating shared antenatal care was obtained. In quantitative studies, generalizability is achieved via statistical sampling procedure, however such sampling procedures are mostly unavailable in qualitative research [34]. In our study, purposive sampling was used for participant selection allowing the selection of relatively small [35] information rich cases for study in depth to illuminate the questions under study [36]. All GPs identified as being antenatal shared care providers in the Geelong, Victoria region by the GP Association of Geelong (n = 175) along with a randomly selected sample of 131 Sydney GPs from the Central Sydney GP Network providing shared care (out of a possible 489), were invited by personal letter to participate. All GPs who replied to the invitation were telephoned by research staff and an interview time was scheduled either via telephone (Sydney GPs) or face to face (Geelong GPs) at a time and location convenient to the GP. They were provided with a plain language statement and consent form either via email or post and all participants provided written, informed consent to participate and have the interview digitally recorded. Consent forms were collected via facsimile from each GP clinic on the day of the interview prior to the interview being conducted. Participating practitioners were reimbursed with a store voucher to the value of one hundred dollars in appreciation of their time. Ethics approval for this study was obtained from the Deakin University and the University of New South Wales Human Research Ethics Committees.

### Data collection and analysis

A descriptive qualitative approach was used in this study to understand more deeply the views of GPs regarding their management of excess GWG. Methods of qualitative description as described by Sandelowski [37,38] were employed. Key design features of qualitative description include maximum variation in sampling, data collection through interviews and qualitative analysis. It also offers a descriptive validity of the situation, that is, an accurate accounting of the events that most people observing the event would agree is accurate. Overall, qualitative interviews provide a flexible approach enabling a probing assessment of a topic. This approach seeks to uncover ideas or concepts that may not have been anticipated at the outset of their research [39].

Individual interviews were employed to enable flexibility and best opportunity to engage with the GP. Interviews were conducted face to face in the Geelong region as this was the main location of the research team and Sydney GPs were interviewed via telephone. Research comparing the reliability and validity of face-to-face and phone interviews has shown a high level of agreement [40]. Semi - structured interviews were employed to ensure all questions (see Table 2) were addressed and this technique enabled comparability of data [41]. The interviews took no more than 30 minutes to complete and were conducted by a researcher trained in qualitative interview techniques. All interviews were transcribed verbatim by an online transcribing company. Thematic analysis was used to assess repeated practices and perspectives across all data. The data were analysed firstly by 6 randomly selected transcripts being read and analysed independently by two of the researchers (PV and JW) to ensure that coding and identification of emerging themes was in agreement. Analysis of the 14 Sydney transcripts was undertaken by KC, and the remaining 14 Geelong transcriptions were analysed by JW. All transcriptions were then re - analysed by PV to ensure consistency. Interview responses made by the GPs were grouped into categories relating to their content. For example, time for first appointment, reasons/thoughts on weighing and not weighing, weight gain advice, referrals, barriers to weighing, support, health consequences etc. Anonymity of participants was maintained through the use of de - identified data.

**Table 2:** Interview Questions

Question		
1.		How many pregnant women would you see on average per year?
2.		In your view, what are the 3 - 5 most important things you think should be covered in a (the first) consultation with a pregnant woman?
3.	(a)	At what point in the pregnancy do women generally present to primary care practitioners?
	(b)	And what about subsequent consultations?
4.		How many consultations would there usually be and what is the focus of subsequent consultations?
5.	(a)	How often are women weighed throughout their pregnancies?
	(b)	Is BMI at first presentation calculated?
	(c)	Is weight trajectory plotted?
6.	(a)	Is advice regarding anticipated gestational weight gain offered?
	(b)	If no, is there a reason for this?
	(c)	Does this take into account BMI at commencement of pregnancy?
7.		If a woman is overweight at first presentation, are you more likely to assess, advise and/or refer for weight management or healthy eating or physical activity education? How would this be done?
8.		What are the triggers that alert you to excess gestational weight gain and increased risk?
9.		In your mind, what do you consider to be the most important implications of overweight and obesity in pregnancy and of weight gain in excess of recommendations?
10.		Do you undertake any assessment of lifestyle behaviours? If so, which lifestyle behaviours do you assess?
11.	(a)	In a perfect scenario, how do you imagine you would be best supported to provide healthy lifestyle advice and support to pregnant women?
	(b)	Do you think that support via the internet, mail or telephone would be useful for weight management advice or healthy lifestyle advice in supporting both yourself and also the pregnant woman?
12.		What sort of information do women mostly seek about their pregnancy and does this ever include weight gain advice?
13.	(a)	Is there much/any interaction with other members of the antenatal team within the practice (such as nurses) regarding weight monitoring or management?
	(b)	If so, how does this happen?

## Results

### Participants

A total of 32 GPs responded to the invitation letter and two of these GPs did not schedule an interview as they were not contactable. Thirty GPs scheduled an interview however 2 GPs were not available at the time of the telephone interview and subsequently did not participate in the study. Twenty eight GPs took part in the interviews, 14 were from Geelong, and 14 from Sydney. GPs were from 22 different clinics within the Greater City of Geelong and metropolitan Sydney and the practices represented a range of socio demographic regions. No GPs withdrew from the study and data from all 28 interviews were included in the analyses. Data was collected until data saturation occurred, that is, when the number of samples have effectively addressed all aspects of the emerging themes and phenomenon with optimal data quality [42].

### Themes

The GPs' responses clustered into five broad themes: (i) GPs own awareness of the issues/identifiable problems of overweight/obesity/excess weight gain; (ii) provision of advice regarding GWG and healthy lifestyle behaviour advice; (iii) attitudes and practices around routine gestational weighing; (iv) practical barriers to management; (v) how GPs feel they could be best supported. Randomly selected quotes are presented by these themes in Table 3.

**Table 3:** Emerging themes and a sample of supporting verbatim quotes

Themes	Verbatim quotes
*Awareness of the issues/identifiable problems of overweight/obesity/excess weight gain*	"But certainly weight is important, and mostly because of gestational diabetes. Really because that impacts on the mother and the baby, and the whole birth outcome."(GP005)
	"Well, gestational diabetes definitely. Possibly pregnancy induced hypertension. And also just complications with delivery because like if, for example they need to have a caesarean, I mean that's going to be really difficult if they're really obese, so, yes, sort of delivery complications as well."(GP026)
	"Well yes there is evidence that it affects the foetus and the wellbeing of the child in future life."(GP016)
	"So I do normally tell the overweight woman about the implication of the long term health problem, plus the implication for young children, because whatever you're doing, your children will be doing the same."(GP018)
	"But obviously children will be obese if mum puts on weight, and keeps going putting on weight or something."(GP017)

*Provision of advice regarding gestational weight gain and healthy lifestyle behaviour advice*	"Actually I'm not quite sure how much is too much. Well certainly gaining to...like 20 kg, that's probably too much."(GP019)
	"I mean we used to work on the sort of one and a half kilograms a month was acceptable. But then when you've got people that are perhaps overweight or obese to start with, we always tried to keep their weight gain at a lot less than that."(GP006)
	"......there's a feeling that exercise during pregnancy may be harmful, particularly in early pregnancy, and to encourage them to keep exercising, I think, is also helpful."(GP006)
	"Actually, when women are pregnant, they're actually very receptive to lifestyle and healthy lifestyle advice."(GP012)

*Attitudes and practices around*	"So I think that weight wasn't a good indicator of maternal or foetal wellbeing, so I think it fell out of favour."(GP005)
	"I noticed that on the shared care antenatal chart established by the hospital the column for weight has disappeared."(GP006)

*routine gestational weighing*	"I'm not sure what the reason why not weighing."(GP008)
	"Weighing (as standard practice) is coming back."(GP005)(GP007)(GP010)
	"Weighing pregnant women is actually very useful when it comes to pre eclampsia."(GP003)

*Practical barriers to weighing*	"I think time is the most significant thing because (I) always have to rush to see patients."(GP019)
	"Space, time, funding, who's going to organise it? So all the organisational and implementation issues."(GP019)

*How GPs feel they could be best supported*	"So, I guess from my point of view it's not just what the doctor says, it's what other health professionals can offer, and motivation."(GP010)
	"If there was a Dietitian attached to this clinic that would be readily available, that would be marvellous, because any woman who I even eyeball to be overweight and therefore at risk of gestational diabetes, I would talk to them about healthy eating in pregnancy and risk of gestational diabetes, and I refer most of my women to this Dietitian."(GP005)
	"......so having access to either a Dietitian or an Exercise Physiologist, or both, that we'd be able to send people to would be good."(GP028)
	"And so you know that people don't remember a lot about a consultation, and so like I say, some written information I think is always good."(GP004)
	"I think that would be a really helpful thing if it was on different aspects of the pregnancy. Because mail - outs or emails, I think that pregnant women are very - they're very centred on doing everything right."(GP011)

### Awareness of the issues/identifiable problems of overweight/obesity/excess weight gain

General Practitioners identified that excess GWG and gestational overweight and obesity adversely affects both the mother and child and the majority of GPs reported that gestational diabetes was one of the most important implications of excess GWG, overweight or obesity in pregnancy. General Practitioners frequently identified pre - eclampsia, hypertension and delivery complications as major implications of excess GWG and maternal overweight and obesity.

Other adverse complications identified infrequently included higher rates of miscarriage, increased rates of unplanned caesarean section, general unspecified obstetric complications, maternal morbidity or mortality, conditions of fatigue, high cholesterol, decreased cardiac fitness, post natal depression and chronic diseases later in life and excessive weight in the post partum period.

General Practitioners recognised varied child health outcomes associated with excess GWG and maternal overweight and obesity including macrosomia, foetal abnormalities and an inability of the practitioner to palpate and examine the baby thus placing the child at higher risk of undetected abnormalities. Few GPs identified child overweight or obesity in the long term as being amongst the most important identifiable problems.

### Provision of advice regarding gestational weight gain and healthy lifestyle behaviour advice

Advice regarding recommended GWG was not consistent and GPs rarely took into account BMI at the start of pregnancy when offering advice. Amount to gain in pregnancy ranged from 8 kg to 15 kg for normal weight women and a small proportion of GPs offered no weight gain advice or offered advice only when asked by the women.

Other than specific nutrient advice and nutrition recommendations for pregnancy, GPs considered general healthy eating advice (in the absence of exercise advice) among the most important topics that should be covered in the initial consultation with the pregnant woman rarely. They infrequently provided exercise advice during pregnancy (in the absence of general healthy eating advice) or mentioned that both general healthy eating advice as well as exercise advice should be given in the first consultation. GPs rarely reported that gestational weight would be among the most important issues to be discussed at the first appointment.

### Attitudes and practices around routine gestational weighing

Weighing practices differed among GPs and most GPs weighed women occasionally throughout their pregnancies. Only a small proportion of GPs weighed women at every visit and few GPs weighed at the first consultation only or never. Attitudes towards weighing varied and there was a clear division in comments provided by the GPs for and against weighing, highlighting a distinct division surrounding usefulness and appropriateness of weighing.

### Practical barriers to management

When GPs were asked about barriers that prevent provision of support to provide healthy lifestyle advice and manage GWG, responses were mixed with approximately one third of GPs mentioning cost to the patient as a financial barrier to provision of additional support, and very few GPs reporting cost as a barrier to hire additional clinicians at the medical practice. Other barriers mentioned by few GPs included lack of space for additional practitioners at the clinic, lack of GP time and short consultation periods and lack of organisational structure within the practice including extensive patient waiting lists as well as patients for weight management being sent to the hospital diabetes clinic and therefore further GP support not being required.

### How GPs feel they could be best supported

The majority of GPs reported being more likely to assess, advise and or refer for weight management to other health practitioners if the woman was overweight at first presentation. Many GPs reported that multidisciplinary support and input from other practitioners would help them feel best supported and most GPs reported that Dietitian support would be preferred. Some GPs suggested support from Exercise Physiologists, Diabetes Educators, Endocrinologists or Midwives would be the preferred support. General Practitioners rarely thought that Personal Trainers could offer useful support.

Few GPs reported that support provided to women via the internet or written resources to reiterate their own advice would be preferred support. When asked specifically whether they thought these avenues of information would be useful in helping convey healthy eating and activity advice and to assist weight management and provide support to the practitioner and also the woman herself, most GPs thought that at least one of these forms of education would be helpful.

## Discussion

This study aimed to examine the perspectives of GPs participating in shared antenatal care regarding GWG and to understand opportunities for primary and secondary prevention of excess GWG. To our knowledge this was the first study to investigate how GPs feel they would be best supported to provide healthy lifestyle advice to pregnant women and healthy GWG management. This study suggests that these GPs had mixed views regarding the management and prevention of excess GWG, demonstrated by their reported recommendations for weight gain in pregnancy, weighing practices and views regarding maternal and child health complications associated with excess GWG being highly varied.

As revealed in this study, there is uncertainty regarding the need or even the desirability to weigh pregnant women in GP consultations. Over a third of GPs in our study either did not weigh at all or weighed only when asked to by the patient. The barriers to weighing women in pregnancy identified in this study were time restraints and uncertainty regarding what advice to give regarding weight gain. Similar findings are reported by Olander et al [3] in a study where focus groups and interviews assessed health practitioners (midwives, social workers and antenatal care centre managers) views regarding GWG [3].

The advice regarding the amount of weight to gain in pregnancy varied widely. This is perhaps not surprising as there are no formal recommendations for GWG in Australia. Women who are informed of their own target for gestational weight gain, however, have been found to be more likely to gain within recommended IOM ranges [43] The inconsistency in approach regarding GWG advice in our study reflects findings from a Dutch study [44] where advice received by 144 pregnant women was assessed. In that study, 12% of participants reported receiving no advice for weight gain from their health care provider, 23% received weight gain advice that was higher than IOM recommendations and 5% received advice that was below recommendations [44]. Further, the majority of women who were overweight or obese pre pregnancy, were advised to gain weight in excess of IOM recommendations [44].

In the non - pregnant population, provision of advice from primary care practitioners incorporating weight gain targets has been found to be an effective strategy in weight management [45]. Potter et al [45] surveyed 366 adult patients from 2 primary care practices and found that one of the components the patients reported most wanting to help them achieve successful weight loss was physician help in setting realistic weight goals. Whilst intervention studies incorporating provision of recommendations for weight gain in pregnancy as part of their intervention component are scarce [45-50] and have produced mixed results, setting realistic weight gain targets in shared antenatal care for women could be a promising step in providing support aimed at preventing excess GWG.

Despite inconsistencies in GWG advice, GPs in this study frequently acknowledged the impact of excess GWG, overweight and obesity on maternal and child health outcomes. Maternal conditions (gestational diabetes, pregnancy induced hypertension, and pre - eclampsia) were more frequently reported as co morbidities of excess GWG than were implications for the child (macrosomia, foetal abnormalities and higher risk of undetected foetal abnormalities). Interestingly, long term child health conditions including offspring overweight and obesity and childhood diabetes were infrequently acknowledged as risks associated with excess GWG.

Recent longitudinal studies suggest that long - term adverse effects on offspring weight and body fatness are important correlates of excess GWG. For example in a prospective study of more than 1000 mother - child pairs, Oken et al [23] reports that mothers with greater GWG had children with greater adiposity at age 3, measured by BMI(OR 1.30, 95% CI: 1.04, 1.62 for each 5 kg) and sub scapular and triceps skin fold thickness(0.26 mm, 95% CI: 0.02, 0.51)[23]. In addition, Reynolds et al [22] examined whether maternal body composition and GWG had persisting effects in 276 offspring at 30 years of age. They found that body fat percentage was higher in offspring of mothers with a greater BMI at the first antenatal visit and that higher offspring body fat percentage was independently associated with higher pregnancy weight gain (7.4%/kg/wk; p = 0.002). Similar significant associations of greater pregnancy weight gain with greater offspring waist circumference, BMI and fat mass at age 30 were also seen [22]. Further, Wrotniak et al [51] reported in a retrospective cohort study of 10,266 mothers that the odds of overweight in offspring at age 7 years increased by 3% for every 1 kg of excess GWG. Prevention of the onset of early childhood overweight is important for public health and is therefore an important consideration for maternal weight gain advice in antenatal care.

Antenatal care has previously been described as an opportune time for healthcare providers to assist women in altering lifestyle affecting weight, nutrition and physical activity [27]. Given that pregnant women are highly motivated to achieve the best outcomes for their child [52], GP advice regarding lifestyle behaviours would ideally be a particularlyimportant component of shared antenatal care. In this study, very few GPs offered both general healthy eating and exercise advice as part of the first consultation. GPs reported a multidisciplinary approach utilising input from allied health professionals would provide the most useful support. Referral to a Dietitian for healthy lifestyle advice and weight management was frequently suggested as the preferred approach. However, there are multiple barriers to the provision of additional health practitioners input which includes increased cost to the patient, to the medical practice itself and to the health system, lack of physical capacity within the medical practice to employ additional practitioners and lack of organisational structure required for additional consulting, all of which were highlighted by GPs in this study.

One alternative approach may be the provision of an Australian government subsidised (Medicare) allied health Enhance Primary Care (EPC) plan, offered to pregnant women for healthy weight and lifetyle management. In the current Australian Medicare system, EPC plans allow for a limited number of GP referred visits per year to allied health practitioners. Patients must be diagnosed with a chronic disease, such as obesity, alongside resulting co -morbidities (for example hypertension or hypercholesterolaemia), and are referred for management of these specific conditions. However pregnancy, subsequent excess weight gain and pregnancy induced co - morbidities does not qualify for management under the government subsidised EPC plan yet long term health benefits and public health savings could potentially justify this scheme for pregnant women.

Limitations of the study included the structure of qualitative data collection through interviews. Assumptions might be made that individual participants have the capacity to reflect and interpret the situation and their actions. Offering the option of telephone interviews to cover accessibility issues may result in interaction not as intimate as face-to-face interviews and does not allow the researcher an opportunity to observe the informant's responses. However interviews do allow participants space to provide information, including historical information, verbally and give the researcher control over line of questioning [53] and this was an efficient method used to access busy GPs in a distant location, from whom we may not have otherwise been able to gain information.

Opportunities to help address some of the existing barriers to employing additional health care providers as a referral point for GPs may lie with more cost effective and time efficient avenues of support. Perhaps GP referral to internet resources, telephone support or written education material that provides useful and reliable healthy lifestyle advice for pregnant women in the management of GWG could be beneficial.

## Conclusion

General Practitioners frequently participating in shared antenatal care identify many adverse maternal and child health outcomes associated with excess GWG, however, management of excess GWG and perspectives of the issue vary widely. From a public health perspective, health care practitioners such as GPs are vital in promoting awareness of the importance of healthy GWG. Strategies to best support GPs in their management of GWG are needed so that best outcomes are achieved for maternal and child health. Further research into how best to support GPs participating in shared antenatal care, along with women during their pregnancy, is needed to help promote healthy GWG.

## Competing interests

The authors declare that they have no competing interests.

## Authors' contributions

PV was responsible for co - ordination of the study, all data collection, contributed to data analyses and drafted the manuscript, KC conceived of the study contributed to its design, contributed to data analyses and helped draft the manuscript, JW contributed to the data analyses and helped draft the manuscript, JO contributed to the recruitment process of GPs in Geelong involved in the study, EDW contributed to the recruitment of GPs in Sydney and to the design of the study. All authors read and approved the final manuscript.

## Pre-publication history

The pre-publication history for this paper can be accessed here:

http://www.biomedcentral.com/1471-2296/12/124/prepub
